# Early Clinical and Radiographic Outcomes of Total Hip Arthroplasty with DELTA ST-C Cup and MINIMA S Stem

**DOI:** 10.3390/medicina59030607

**Published:** 2023-03-19

**Authors:** Nikolaos Christodoulou, Emmanouil Volonakis, Karatzas Voutsas, Konstantinos Raptis, Christos Koutserimpas

**Affiliations:** 1 Department of Orthopedics, Athens Medical Group, Psychicko, 11525 Athens, Greece; 2 Department of Orthopedics and Traumatology, “251” Hellenic Air Force General Hospital of Athens, Kanellopoulou Av, 11525 Athens, Greece

**Keywords:** acetabulum fixation, bone preservation, minimally invasive surgery, press fit, total hip arthroplasty, threaded cup, short stem

## Abstract

*Background and Objectives*: The uncemented threaded DELTA ST-C cup was introduced in last few years. It has a hemispheric shell, consisting of Ti6Al4V titanium alloy. The MINIMA S stem was developed according to the principles of proximal-loading and extended metaphyseal geometry applied to a short stem. The purpose of the study was to assess the clinical and radiographic outcomes and the short- and mid-term survivorship of the DELTA ST-C cup and MINIMA S stem in patients undergoing total hip arthroplasty (THA). The present is the first study to report outcomes and implant survivorship of the DELTA ST-C cup coupled with the MINIMA S femoral stem. *Materials and Methods*: The present study is a retrospective observational cohort study of a prospectively maintained database, evaluating clinical outcomes and implant survivorship in 95 patients undergoing THA with the MINIMA stem coupled with the DELTA ST-C cup with at least a 3-year follow-up. The clinical evaluation was assessed with a change in the Harris hip score (HHS), while the radiographic evaluation included anteroposterior views of the pelvis and lateral views of the affected hip. *Results*: The enrolled population’s mean age was 69.3 years and most patients were female (64%). The MINIMA S standard stem was implanted in 68 patients (72%), the lateralized stem was implanted in 27 (28%), and the mean acetabular inclination was 48.2°. The HHS improved significantly from the preoperative value (median 46, IQR: 38–55), already at 1 month after surgery (median 76, IQR: 66–77), reaching excellent results at 1 year and 3 years postoperatively (median 96, IQR: 91–100). X-rays demonstrated good implant stability and biomechanics parameter restorations revealed no sign of subsidence, and the presence of radiolucent lines greater than 2 mm in the short stem area in five cases and in the acetabulum in one were not clinically significant. No revisions have been performed so far. *Conclusions*: The MINIMA S stem coupled with the DELTA ST-C cup demonstrated very good clinical and radiological results with a significant increase of the Harris hip score at short- and mid-term follow-up. This is the first study evaluating the DELTA ST-C cup, showing promising outcomes during the study’s follow-up. The MINIMA S stem has been evaluated in a very few studies. However, the combination with this particular cup had not yet been studied. The design of the stem and the cup ensures primary stability and excellent early term outcomes, moreover the study demonstrates extraordinary implant survivorship, equal to 100%.

## 1. Introduction

Total hip arthroplasty (THA) represents a very successful, widely performed procedure for patients with a variety of painful conditions involving the hip joint. THA may alleviate pain and restore function, as well as improve quality of life. Nowadays, patients’ expectations following THA have increased, taking into account quality of life issues and recreational interests [1,2].

Cementless acetabular cups were introduced in the 1970s to overcome “cement disease”, progressive bone resorption related to particulate debris at the cement-bone interface, which is considered to be the main cause of failure of cemented polyethylene (PE) cups due to aseptic loosening [3,4].

Primary fixation of the acetabular components may still be achieved in various ways, by press-fit or by threads, with or without screws [4]. Although the press-fit cups are more commonly used nowadays, experimental studies have shown that threaded cups have a higher primary stability compared to press-fit cups with respect to lever-out forces and interface micromotions [5]. Another reason why threaded cups are not so popular among hip surgeons could be the fact that the first generation threaded cup had a smooth surface that led to failure, because the implant was never integrated [4,5]. 

The new generation of threaded cups has shown a high primary stability which is essential for a good subsequent osteointegration and, thus, also for long-term secondary stability [5,6]. The primary stability of these components is based on a press-fit situation by threads screwed into the bone, and it depends on several factors, such as the design of the rim, the cup’s geometry, the implant material, as well as the surface finishing [5,7].

The new threaded acetabular cups are composed of a metallic hemispherical shell with a rough surface to improve the osseous integration and the long-term survivorship. In addition, modular metal, ceramic and modified polyethylene inserts are available [7].

The DELTA ST-C cup (Lima Corporate, Villanova di San Daniele del Friuli, Udine, Italy) has a hemispheric shell, consisting of Ti6Al4V titanium alloy and is provided with six helicoids, ensuring optimal initial stability and supporting torsional loads weighing in the equatorial area, without interrupting the continuity of the bone wall. It also allows for the complete support in the acetabular seat so the load is transferred to the entire surface of the subchondral bone without generating overloads in the proximity of the helicoids, thus allowing for considerable bone saving [8]. The DELTA ST-C cup was recently developed and no studies exist so far that evaluate the implant’s survivorship or implant-related complications.

Femoral cementless fixation in THA has been proven clinically and radiologically to have excellent long-term results [9]. Considering that more THAs are performed in younger and more active individuals, over the last two decades, a new total hip replacement philosophy, called “tissue sparing surgery”, has become more popular [9,10]. The principles consist of reduced bone and soft tissue aggression; therefore, new conservative short femoral prostheses have been designed as alternatives to conventional stems. These less invasive designs are less than 12 cm long, and are commonly named “short stems”. Nevertheless, this term refers to a heterogeneous group of stems that present differences in terms of design, biomechanics and load-bearing properties [10]. Based on the bone preservation philosophy, short-stem total hip arthroplasty (THA) has primarily been recommended for young and active individuals [10]. However, there may be benefits for elderly patients, given the less invasive operative technique due to the short-curved implant design. Nevertheless, femur morphology may prohibit the implantation of an uncemented short stem. According to the Dorr classification, femoral type A (cortices seen on both anteroposterior and lateral X-ray views) and B (thinning of the posterior cortex on lateral X-ray views) permit the implantation of uncemented stems, while type C (thinning of the cortices on the anteroposterior and lateral X-ray views) seems to be an indication for the implantation of cemented femoral prostheses [11].

**Figure 1:**
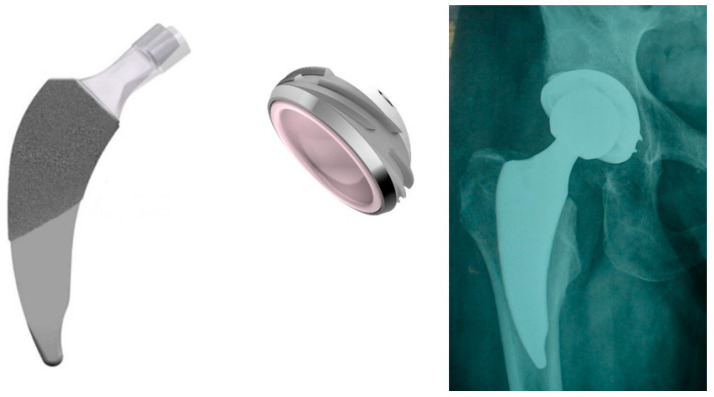
The MINIMA S stem coupled and the DELTA ST-C cup, along with a postoperative anterior-posterior X-ray view.

MINIMA S stem (Lima Corporate, Villanova di San Daniele del Friuli, Udine, Italy) was developed according to the principles of proximal-loading and extended metaphyseal geometry applied to a short stem. Its design, mainly its rectangular cross section, provides anti-rotational properties and allows for achieving metaphyseal fitting and optimal primary fixation with metaphyseal loading. Secondary fixation is achieved due to the anatomic design (maximized contact) and the proximal porous titanium coating [12]. Few studies reporting on the radiological findings and clinical results with short-term follow-up of the MINIMA S stem exist so far. It is of note that the combination with the DELTA ST-C cup has not been studied yet. It is of importance to report the outcomes of this new implant combination, including the short-term outcomes, so that surgeons may have evidence-based information for using these implants in THA (Figure 1).

The present study evaluates early radiographic findings and the functional recovery in terms of implant stability and survivorship of primary hip arthroplasty with MINIMA S stem coupled with the DELTA ST-C cup with at least a three-year follow-up. The present cohort study is the first one to report outcomes and implant survivorship of the DELTA ST-C cup coupled with the MINIMA S femoral stem.

## 2. Methods

This is a retrospective observational cohort study of a prospectively maintained database, evaluating the early clinical outcomes and implant survivorship in patients undergoing THA with the MINIMA S stem coupled with the DELTA ST-C cup. 

**Figure 2:**
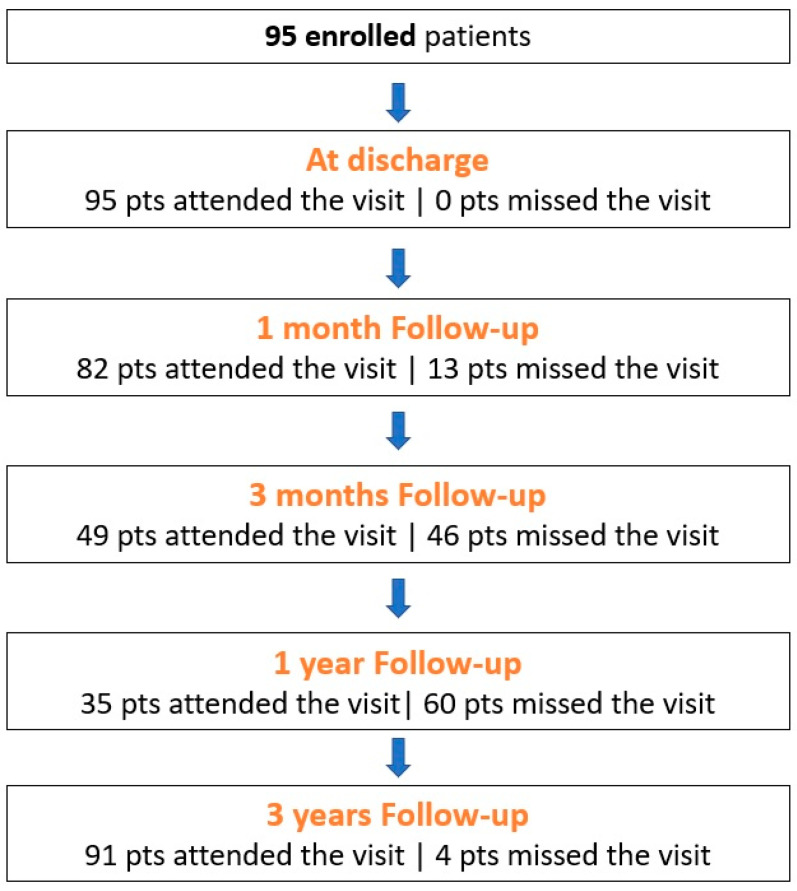
Study flow: up to 3 years of follow-up. It is of note that at the final follow-up, the four patients who missed the visit had passed away. Pts: patients.

A total of 95 patients undergoing primary THA with the DELTA ST-C cup in combination with the MINIMA S stem at the Psychiko Clinic (Athens Medical Group, Athens, Greece) from January 2017 to December 2018 were enrolled in the study (Figure 2). Patients were eligible if they were older than 18 years, suffering non-inflammatory degenerative joint disease, such as osteoarthritis, avascular necrosis, rheumatoid arthritis, post-traumatic arthritis, femoral neck fractures and congenital or acquired deformity. 

Local or systemic infections, hypersensitivity to used materials, persistent acute or chronic osteomyelitis, significant neurological or musculoskeletal disorders that may affect the functional outcome, known metabolic disorders that could impair fixation and stability of the implant, as well as vascular or nerve disease affecting the limb were exclusion criteria. Revision cases were also excluded. Furthermore, patients classified as Dorr type C femurs, upon initial evaluation, were excluded as well.

Demographics, intensity of daily activity (intensive, normal, sedentary) and work status (active worker, retired) were recorded at the first visit, while a pre-operative assessment included radiographic and clinical evaluation, including the Harris hip score (HHS) score. The HHS evaluates the results of hip surgery and is intended to assess various hip disabilities and methods of treatment in an adult population. The maximum score possible is 100, while outcomes may be interpreted as <70 = poor result; 70–80 = fair, 80–90 = good, and 90–100 = excellent [13].

The clinical evaluation was assessed as a change in the HHS from the baseline (preoperatively) up to the final follow-up. This score was recorded at the following time-points: pre-operatively and 1 month, 3 months, 1 year, as well as 3 years post-operatively. 

**Figure 3:**
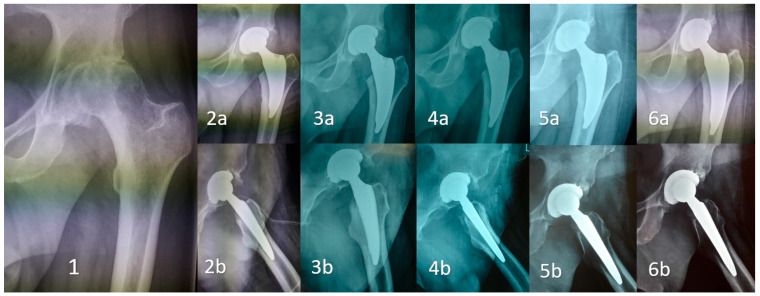
Radiographic evaluation throughout the study. (1) Preoperative anteroposterior (AP) view. (a) AP view, (b) lateral view. (2) postoperative period, (3) One-month follow-up, (4) 3-month follow-up, (5) 1-year follow-up and (6) 3-year follow-up.

The radiographic evaluation included anteroposterior (AP) views of the pelvis and lateral views of the affected hip, and was performed in the pre-operative phase and post-operatively at discharge, and after 1 month, 3 months, 1 year and 3 years. Radiographic evaluation and stability assessment of the DELTA ST-C cup were studied as the rate of symptomatic radiolucent lines, a loosening and subsidence ≥ 2 mm from discharge (baseline) up to the 3-year follow-up. Furthermore, a radiographic evaluation and stability assessment of the MINIMA S stem were studied as the rate of symptomatic radiolucent lines, loosening and subsidence ≥ 2 mm from discharge (baseline) up to 3-year follow-up. Additionally, survival rate of the implant was performed and any adverse device events or device-related complications were detected and recorded. Radiographic evaluation of the acetabular component was performed with the use of DeLee and Charnley zones. The DeLee and Charnley system applies to the acetabular cup on anteroposterior radiographs. It divides the acetabulum into three equally large zones (zone I: superior, II: middle, III: inferior) [14]. Radiographic evaluation of the femoral component is performed with the use of Gruen’s zonal system (consecutive zones 1–7 at the AP X-ray views; zone 1: greater trochanter, zone 4: stem tip, zone 7: lesser trochanter) [15]. Figure 3 depicts the radiographic evaluation of a case throughout the study period.

All patients, under subarachnoid anesthesia, underwent THA with the modified anterolateral minimal invasive approach (ALMIS) in the lateral position [16,17]. They received chemoprophylaxis 1-h pre-operatively, including 1 gram of vancomycin. Then, they were commenced on thromboprophylaxis 12 h after the operation, with tinzaparin, and this was continued for 30 days. It should be noted that vancomycin is used in our institution for prophylaxis in total joint surgery, due to the recently frequent isolation of methicillin resistant *S. epidermitis*.

Physiotherapy aimed for early mobilization, coordination, stability and strengthening of the hip’s muscles, especially abductors, in order to improve the range of motion and walking performance. Physiotherapy was initiated from the day of the surgery, including walking training (sit to stand, crutches and stairs), as well as bed exercises, including isometric quadricep strengthening. The use of walking aids was recommended during the first 6 weeks, while abductor exercises were strongly recommended. 

Comparison of the quantitative variables between the follow-up time-points were evaluated by Wilcoxon tests. A McNemar test was used to compare the binary matched-pairs data. Spearman correlation coefficients were calculated between HHSs and some variables of interest. A *p*-value less than 0.05 was considered statistically significant and 95% confidence intervals were provided where needed. STATA13 statistical software was used for every statistical computation.

This study was conducted in accordance with the ethical principles that have their origin in the “Declaration of Helsinki, Ethical Principles for Medical Research Involving Human Subjects” (October 2008), in compliance with the requirements stated by EN ISO 14155 (Clinical investigations of medical devices for human subjects–Good clinical practices) and European and national regulations. 

The study has also been approved by the institution’s (Athens Medical Group, Psychicko, Athens, Greece) bioethics committee (ref: 23/26-3-2020), while informed written consent was received from all participants. Furthermore, the study has been included in the registry of clinical trials as NCT05014113.

## 3. Results

A total of 95 patients undergoing THA with the MINIMA S stem coupled with the DELTA ST-C cup, between January 2017 and December 2018, were included in the study.

Table 1 highlights the main demographics of the studied population. Sixty-four percent of the enrolled population were women, the sample’s mean age was 69.3 years [standard deviation (SD) = 10.4] and the median BMI was 27.4 kg/m^2^ (25.2–30.2 IQR). Regarding the level of activity, most patients were retired (52; 55%) and led mostly sedentary (35; 37%) and normal (33; 35%) lifestyles, while 27 patients reported an intensive daily lifestyle (28.4%).

Patients were mainly affected by primary osteoarthritis (67; 70.5%). Preoperatively, most of them suffered from severe pain with every movement (18 patients; 19%) and occasionally (57; 60%). Non-steroid anti-inflammatory drugs (NSAIDs) were used regularly in 12 (13%) cases and occasionally in 75 (79%) cases, as pain relief management.

From the intraoperative information, in most cases (89.5%) the acetabular bone stock quality was good, while for the remaining (10.5%), it was characterized as osteoporotic. In 88% of patients, the femoral bone stock quality was considered normal and in 12% of cases, it was considered osteoporotic. The mean hospital stay was 2.6 days (SD = 0.8).

The MINIMA S standard stem was implanted in 68 patients (72%), the lateralized stem was implanted in 27 (28%) and the most common cup diameter was 50 mm (30; 31.6%), followed by that of 52 mm (16; 16.8%).

Regarding the clinical outcomes, the change in the HSS values is depicted in Table 2. More specifically, the median preoperative HSS was 46 (38–55 IQR), there was a significant increase 1 month after surgery, with median values reaching 76 (66–77 IQR). A median HHS at 3 months after the operation was 93 (90–96 IQR) and at the 1 and 3-year follow-up, the median HHS was 96 (91–100 IQR).

Regarding the preoperative radiographic evaluation, a mean of 1.8 cm (SD = 3.4) leg length discrepancy was recorded. In 14 cases (14.7%), a valgus deformity was observed and a varus deformity was observed in six (6.3%).

In the immediate postoperative radiographic evaluation, the sizing of the stem was found to be normal in most cases (86; 90.5%) and undersized in the rest (9; 9.5%). The position of the stem was found to be neutral in 60 cases (63.2%), at the valgus in 33 (34.7%) and at the varus in two (2.1%).

The postoperative radiographic analysis of the femoral stem is depicted in Table 3. At the 3-month follow-up, in three cases (3.2%), radiolucent lines ≤2 mm were observed and at the 3-year follow-up, in 45 cases (47.3%), radiolucent lines ≤2 mm were noted; the Gruen zones mostly affected were zones 3 (16 cases; 35.6%), 4 (14; 31.1%), 5 (6; 13.3%) and 10, 11 and 12 (three each, 6.7%). At the 3-year follow-up, in five cases (5.3%), lines >2 mm were evident, mostly affecting Gruen zones 1 (3 cases; 60%) and 2 (2; 40%). No cases of subsidence were found. Furthermore, at the 3-year follow-up, sixteen cases (16.9%) of cortical hypertrophy were recorded, mainly affecting Gruen zones 2 (eight cases; 50%), 3 and 5 (four cases each; 25%). 

**Figure 4:**
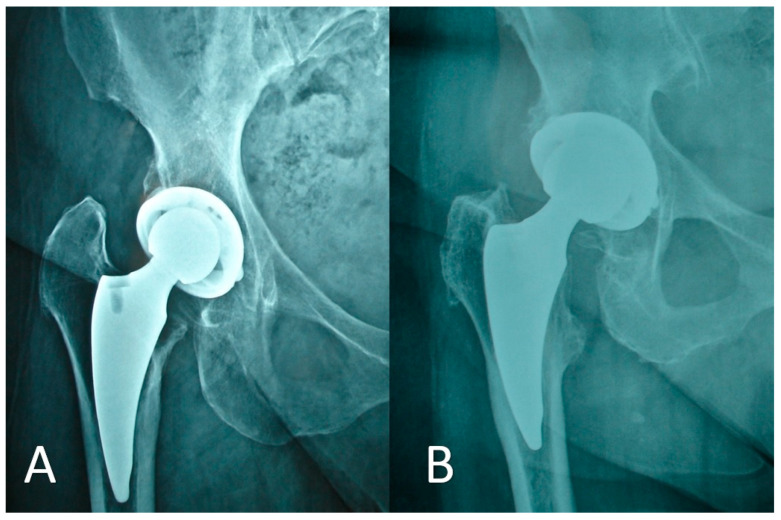
(**A**) DeLee and Charnley zone 2: a >2 mm radiolucency of the acetabular component. (**B**) Gruen zone 3: a femoral cortical hypertrophy.

The postoperative radiographic analysis of the DELTA ST-C cup is highlighted in Table 4. The mean acetabular inclination was radiographically measured to be 48.2° (SD = 5.1). The acetabular components remained stable during the entire study period. More specifically, no cases of acetabular component migration were recorded. In one (1%) case, at the final 3-year follow-up, a >2 mm radiolucent line in DeLee and Charnley zone 2 (Figure 4) was revealed. This patient had no signs or symptoms of implant loosening. Furthermore, in eight cases (8.5%), at the 3-year follow-up, a <2 mm radiolucent line was noted.

The presence of a radiolucency > 2 mm was evident at the final follow-up in five (5.6%) cases in the femoral zone (*p*-value = 0.06, when compared to the immediate postoperative period) and in one (1%) case in the acetabular zone (*p*-value = 1). 

**Figure 5:**
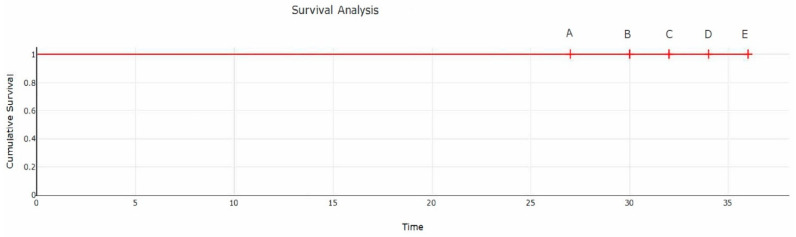
Survival analysis. Four patients (points A, B, C, D; 27, 30, 32, 34 months) had passed away before the final follow-up at 36 months (point E). Points A (27 months), B (30), C (32) and D (34) represent the time points when these patients passed away, and up to that point no implant failure was recorded.

A total of 91 patients attended the last 3-year follow-up visit. The four missing patients had passed away, all shortly before the last (3-year) follow-up. No revisions or implant failure were mentioned by their relatives. Since no revisions or implant failures had occurred, the MINIMA S stem coupled with the DELTA ST-C cup survivorship at 3 years after surgery was 100% (Figure 5).

During the study period, no evidence of loosening, infection, hardware failure or dislocation were observed, while one case of deep venous thrombosis was recorded.

## 4. Discussion

New generation threaded cups have already shown a high primary stability, representing an essential factor for good subsequent osteointegration and, hence, also for long-term secondary stability [5,6,18,19,20]. The DELTA ST-C cup is a cementless primary implant with screw-in fixation, which is characterized by a hemispheric design and is also provided with helicoids designed to guarantee optimal initial stability and to support torsional loads weighing in the equatorial area, without interrupting the continuity at the bone wall and encompassing considerable bone saving [8]. The cup is manufactured in titanium alloy (Ti6Al4V, ISO-5832-3) with double coating, a porous titanium layer and an additional hydroxyapatite layer, to further enhance the mechanical friction with the acetabular wall and stimulate the osteointegration process [8].

MINIMA S femoral stems are made of forged Ti6Al4V. The proximal or metaphyseal region has a VPS porous titanium coating (approximately 200 µm) to guarantee and improve the primary and secondary stability. The distal surface has a macro roughened surface that is obtained by blasting with a controlled flow of corundum particles [12]. Following the findings that the diaphyseal tract of a traditional femoral component was no longer necessary to implant stability, once proximal fixation was achieved, the MINIMA S femoral stem was created as part of the efforts made to develop short femoral solutions [21,22,23,24,25,26].

The purpose of this study was to evaluate the early functional and radiographic outcomes, in terms of implant stability and survivorship, of primary hip arthroplasty with the MINIMA S stem coupled with the DELTA ST-C cup. It is of note that this is the first such study with a 3-year follow-up for these implants. 

The present study has revealed significant improvement in the clinical score (HHS), and during the study period, no evidence of loosening, infection, hardware failure or dislocation was observed in patients treated with THA, with the modified ALMIS approach using the MINIMA S short stem and the DELTA ST-C cup.

The outcomes following THA have also been associated with the individual’s physical activity and BMI. In the present cohort, most patients were retired (52; 55%) and the majority were overweight (34; 46.7%). Stresses arise more in overweight patients and this could lead to implant breakdown in the long-term [27].

Regarding the MINIMA S stem, the results of this study are aligned with similar studies in the literature with a shorter follow-up or studies regarding other short stems with a similar design [28,29,30,31]. Radiographic evaluation demonstrated good implant stability. Additionally, no signs of subsidence were revealed, the presence of radiolucent lines greater than 2 mm was observed in only five cases, and these events were not statistically significant. Furthermore, 16 cases of cortical hypertrophy at 3 years after surgery were reported without affecting the clinical outcomes. 

Lidder et al., in a systematic review, analyzed different short stems and reported improvement in the HHS from a mean preoperative score of 46 (0 to 100) to a mean postoperative score of 92 (39 to 100) [29]. This study also showed a statistically significant HHS improvement. There are two studies so far in the literature that evaluate the MINIMA S stem with up to two years of follow-up, and these report similar outcomes to the present study [30,31]. In particular, all three studies (including the present one) have demonstrated a stable and well osseointegrated stem with no complications, such as subsidence, fractures, dislocations or infections. More particularly, Drosos et al. initially evaluated 61 patients with a mean age = 56 years, who received a MINIMA S stem with a mean 2.8-year follow-up [31]. The cohort in our study had a mean age of 69.3 years. Drosos et al. reported no major complications, such as revision, periprosthetic fracture, dislocation or infection, and these results are similar to ours [31]. This raises the issue of short-stem indication. It has been reported that these types of femoral stems are more suitable for younger, more active patients. Nevertheless, this is not absolute, as shown by our reported short-term outcomes. It is of note that a press fit cup was used in Drosos et al.’s study.

It should be noted that some cases of bone remodeling were recorded in this study. This should not be considered an issue, since the presence of bone remodeling in short femoral stem cases has been reported in the literature during early follow-up and it is not correlated with inferior clinical outcomes [31,32,33]. Cortical hypertrophy is reported in 14–60% of patients receiving short femoral stems at early radiological follow-up. This may be associated with mild thigh pain, resolving after 1 year. Cortical hypertrophy mainly affects Gruen zones 2, 3 and 5.

Threaded cups have exhibited biomechanical advantages when compared to press fit cups, but they are not commonly used in every-day clinical practice [7]. The main reason seems to be that between 1970 and the 1980s, first generation threaded cups with a smooth surface were widely used and had high failure rates, due to the lack of bony ingrowth [6,7,19]. The main concerns were early loosening, cup breakage and soft tissue damage, as well as difficulty in removing the cup in revision cases [7]. Second and third generation threaded cups have a porous coating, allowing for bony ingrowth [6,19]. Nevertheless, they are not considered the most “popular” choice of cup implant. 

Porous coated threaded cups have shown promising results at early, as well as intermediate, follow-up. Gala et.al. compared 150 threaded porous coated acetabular cups to 150 press-fit cups, and revealed no statistically significant differences between the two groups regarding all of the parameters, including the implant’s survivorship and patients’ clinical outcomes, at a mean follow-up of 52.5 months [7]. Manley et al. radiographically showed in follow-ups of 5 years, that 99% of the porous coated threaded cups were stable with osseous ingrowth, as indicated by the absence of radiolucency at the interface and the absence of migration within the acetabulum [34]. In our study, one (1%) case at the final 3-year follow-up revealed a >2 mm radiolucent line in DeLee and Charnley zone 2. It is of note that this patient had no signs or symptoms of implant loosening. Moreover, in eight cases (8.5%), at the 3-year follow-up, a <2 mm radiolucent line was recorded.

Regarding the long-term follow-up of porous threaded cups, favorable outcomes have also been reported. Schmolders et al. conducted a retrospective study where threaded acetabular cups were implanted with cementless femoral stems in 100 patients younger than fifty years of age. The clinical outcomes showed that the mean HHS improved from 45 pre-operatively to 98 at the time of last follow-up. Moreover, the overall survival rate of the implant was 96.8% at the ten-year follow-up [35]. Reikeras et al., in a comparative study, showed that porous-coated threaded cups had a 91% survival rate at 16 years, compared to the porous-coated press-fit cup with a 74% survival rate [36]. Furthermore, McLaughlin et al. reported a survival rate of 94% for threaded cups at 25 years of follow-up [37]. In our reported cohort, the preoperative (baseline) mean HHS was 46, which finally reached 96 at the 3-year follow-up.

Regarding the DELTA ST-C cup, the results of the present study have shown good radiological findings, as indicated by the absence of radiolucency at the interface and the absence of migration within the acetabulum, as well as favorable clinical outcomes. Furthermore, no intraoperative fracture or postoperative implant-related complications were recorded. Threaded cups may have different designs in terms of implant geometry, as well as helicoids. Such cups have already been successfully used in treating patients suffering from primary osteoarthritis, as well as patients with dysplastic hips [17,37,38]. However, there are not many studies evaluating the outcomes of threaded cups.

It should be noted that the initial data provided in this study showed that no major complications were recorded in this cohort and no revisions have been performed so far. Moreover, at short-term follow-up, it seems that these implants had favorable outcomes. However, longer follow-up is of utmost importance for evaluating this implant. Nevertheless, many major complications in THA, such as intraoperative fractures, prosthetic joint infections due to inoculation, allergic reactions to the prostheses and dislocations may occur shortly after the operation. It is of utmost importance for the surgeon to know that the used implant has not been associated with such adverse effects. The early failure of implants would prohibit their use in clinical practice. Longer follow-up is needed in the future for evaluating the materials’ long-term behavior.

The present study has some limitations. It is a single center study, it has a retrospective nature and does not encompass a control group. Due to its retrospective nature, not all patients were followed-up at each time point (as shown in Figure 1). Comparison with other implant combinations, including press-fit cups with similar short stems was also not possible. Furthermore, the study reports early results of hip prostheses and more follow-up is needed for the evaluation of these implants’ survivorship and implant-related complications. Nevertheless, it is of note that this is the first study reporting the results of the MINIMA S stem coupled with the DELTA ST-C cup with a minimum of 3-year follow-up. Hence, even short-term outcomes of a new implant combination should be reported, in order for clinicians to have evidence-based data to proceed with using these implants in THA.

## 5. Conclusions

The MINIMA S stem coupled with the DELTA ST-C cup demonstrated very good early clinical and radiological results, with a significant increase in the HHS at short- and mid-term. The design of the stem and the cup ensures primary stability and excellent early term outcomes. Moreover, the study demonstrated extraordinary implant survivorship, equal to 100% at the 3-year follow-up. These results show that this implant combination may be safely used for primary hip arthroplasty, and surgeons should expect favorable outcomes. It should also be noted that an additional aim of this study was to reveal that threaded cups remain a viable option for surgeons who have experience in the use of such implants. However, further research with more follow-up is of utmost importance for evaluating the implants’ survivorship and implant-related complications in the long-term. Additionally, comparative studies evaluating different implant combinations in the long-term would be also of importance.

## Figures and Tables

**Table 1:** Demographics of the studied population. BMI: body mass index.

Demographic/Anthropometric		Total Cohort (n = 95)
Age (years), mean (SD)		69.3 (10.4)
Range (min–max)		42–91
Age classes, n (%)	<60 years	31 (32.6)
	60–70 years	35 (36.8)
	>70 years	29 (30.5)
Sex, n (%)	Females	61 (64.2)
	Males	34 (35.8)
Weight (Kg), median (IQR)		75.5 (67–86)
N–range (min–max)		92 (51–145)
Height (cm), median (IQR)		165 (160–172)
N–range (min–max)		92 (150–190)
BMI (kg/m^2^), median (IQR)		27.4 (25.2–30.2)
N–range (min–max)		92 (17.7–42.2)
BMI classes, n (%)	Underweight (<18.5)	1 (1.1)
	Healthy weight (18.5–24.9)	20 (21.7)
	Overweight (25–<30)	43 (46.7)
	Obese (≥30)	28 (30.4)

**Table 2:** Change in the Harris hip score (HHS) values between each time-point compared to the baseline (preoperative).

		1 Month vs. Baseline		3 Months vs. Baseline		1 Year vs. Baseline		3 Years vs. Baseline	
	Baseline	1 Month	*p*-Value	3 Months	*p*-Value	1 Year	*p*-Value	3 Years	*p*-Value
HHS values, median (IQR)	46 (38–55)	76 (66–77)	<0.0001	93 (90–96)	<0.0001	96 (91–100)	<0.0001	96 (94–100)	<0.0001

**Table 3:** Radiographic analysis of the femoral MINIMA S stem during follow-up. Postop: postoperative.

Postoperative Analysis—Femoral Side						
		Postop	1 Month	3 Months	1 Year	3 Years
Radiolucent Lines						
Assessment not available		0 (0%)	14 (14.7%)	46 (48.4%)	60 (63.2%)	6 (6.3%)
	None	95 (100%)	80 (84.2%)	46 (48.4%)	24 (25.2%)	39 (41.1%)
	≤2 mm	0 (0%)	1 (1.1%)	3 (3.2%)	10 (10.5%)	45 (47.3%)
	>2 mm	0 (0%)	0 (0%)	0 (0%)	1 (1.1%)	5 (5.3%)
Stress Shielding						
	Assessment not available	0 (0%)	14 (14.7%)	46 (48.4%)	60 (63.2%)	6 (6.3%)
	None	95 (100%)	81 (85.3%)	48 (50.5%)	28 (29.5%)	38 (40%)
	Proximal femur	0 (0%)	0 (0%)	1 (1.1%)	7 (7.3%)	51 (53.7%)
Cortical Hypertrophy						
	Assessment not available	0 (0%)	14 (14.7%)	46 (48.4%)	60 (63.2%)	6 (6.3%)
	None	95 (100%)	81 (85.3%)	48 (50.5%)	33 (34.7%)	73 (76.8%)
	Present	0 (0%)	0 (0%)	1 (1.1%)	2 (2.1%)	16 (16.9%)
Subsidence						
	Assessment not available	0 (0%)	14 (14.7%)	46 (48.4%)	60 (63.2%)	6 (6.3%)
	Absent (<5 mm)	95 (100%)	81 (85.3%)	49 (51.6%)	35 (36.8%)	89 (93.7%)
	Present (>5 mm)	0 (0%)	0 (0%)	0 (0%)	0 (0%)	0 (0%)

**Table 4:** Radiographic analysis of the DELTA ST-C cup during the follow-up period.

Postoperative Analysis—Acetabular Side						
		Postop	1 Month	3 Months	1 Year	3 Years
Radiolucent Lines						
	Assessment not available	0 (0%)	14 (14.7%)	46 (48.4%)	60 (63.2%)	6 (6.3%)
	None	95 (100%)	81 (85.3%)	49 (51.6%)	34 (35.8%)	80 (84.2%)
	≤2 mm	0 (0%)	0 (0%)	0 (0%)	1 (1%)	8 (8.5%)
	>2 mm	0 (0%)	0 (0%)	0 (0%)	0 (0%)	1 (1%)
Migration						
	Assessment not available	0 (0%)	14	46	60	6
	Absent (<2 mm)	95 (100%)	81 (100%)	49 (100%)	35 (100%)	89 (100%)
	Present (>2 mm)	0 (0%)	0 (0%)	0 (0%)	0 (0%)	0 (0%)

## Data Availability

Not applicable.
